# Salinomycin Activates AMP-Activated Protein Kinase-Dependent Autophagy in Cultured Osteoblastoma Cells: A Negative Regulator against Cell Apoptosis

**DOI:** 10.1371/journal.pone.0084175

**Published:** 2013-12-17

**Authors:** Lun-qing Zhu, Yun-fang Zhen, Ya Zhang, Zhi-xiong Guo, Jin Dai, Xiao-dong Wang

**Affiliations:** The Center of Diagnosis and Treatment for Children’s Bone Diseases, the Children’s Hospital Affiliated to Soochow University, Suzhou, Jiangsu, China; Wayne State University, United States of America

## Abstract

**Background:**

The malignant osteoblastoma has poor prognosis, thus the search for novel and more efficient chemo-agents against this disease is urgent. Salinomycin induces broad anti-cancer effects both *in* *vivo* and *in* *vitro*, however, its role in osteoblastoma is still not clear.

**Key Findings:**

Salinomycin induced both apoptosis and autophagy in cultured U2OS and MG-63 osteoblastoma cells. Inhibition of autophagy by 3-methyladenine (3-MA), or by RNA interference (RNAi) of light chain 3B (LC3B), enhanced salinomycin-induced cytotoxicity and apoptosis. Salinomycin induced a profound AMP-activated protein kinase (AMPK) activation, which was required for autophagy induction. AMPK inhibition by compound C, or by AMPKα RNAi prevented salinomycin-induced autophagy activation, while facilitating cancer cell death and apoptosis. On the other hand, the AMPK agonist AICAR promoted autophagy activation in U2OS cells. Salinomycin-induced AMPK activation was dependent on reactive oxygen species (ROS) production in osteoblastoma cells. Antioxidant n-acetyl cysteine (NAC) significantly inhibited salinomycin-induced AMPK activation and autophagy induction.

**Conclusions:**

Salinomycin activates AMPK-dependent autophagy in osteoblastoma cells, which serves as a negative regulator against cell apoptosis. AMPK-autophagy inhibition might be a novel strategy to sensitize salinomycin’s effect in cancer cells.

## Introduction

The malignant osteoblastoma is the most malignant bone tumor with poor prognosis. Although this disease is mostly seen in teenagers, it is typically diagnosed at an advanced stage when surgery can no longer remove the entire tumor [1]. In order to ensure a cure, it is important to develop more effective chemo-agents [2]. However, the malignant osteoblastoma is among the most intrinsically resistant tumors to multiple chemotherapeutic drugs [3]. As such, the search for novel chemo-agents is urgent [2]. 

A recent high-throughput screening study has demonstrated that salinomycin selectively kills breast cancer stem cells from tumorspheres and to inhibit tumor growth in mice [4]. Since, salinomycin has been investigated as an anti-cancer agent [4,5]. Meanwhile, studies have shown that salinomycin inhibits the growth of various immortalized cancer cells both *in vivo* and *in vitro* [6–9]. The mechanisms of salinomycin-induced anti-cancer efficiency are not fully understood; Although Wnt suppression [7], p-glycoprotein inhibition [5] and reactive oxygen species (ROS) production have been proposed [8]. In the current study, we aim to investigate the potential anti-cancer ability of salinomycin against osteoblastoma cells, and to study the underlying mechanisms.

AMP-activated protein kinase (AMPK), the conserved cellular energy sensor, plays a vital role in energy homeostasis maintenance [10–12]. In addition, AMPK is also important for the regulation of many other cellular processes including cell growth, protein synthesis, apoptosis, and autophagy [10,12]. Autophagy is a highly conserved process for degradation and recycling of cytoplasmic components in lysosome, it is important for maintaining cellular structure and function [13–16]. In cancer cells, autophagy is generally known as a pro-survival and chemo-resistance factor, probably due to its anti-apoptosis ability [13,17,18]. Thus, autophagy inhibition has been proven to be a useful strategy for chemo-sensitization [17–19]. Activation of AMPK induces autophagy through at least two following mechanisms: 1. By phosphorylating and activating of Ulk1, the autophagy initiator [20,21]; 2. By inhibiting of the mammalian target of rapamycin (mTOR) complex 1 (mTORC1), the autophagy suppressor [20,21].

In the current study, we observe that salinomycin induces both autophagy and apoptosis in cultured osteoblastoma cells. AMPK activation by salinomycin mediates autophagy induction, which serves as a negative regulator against cell death and apoptosis. Our results indicate that AMPK/autophagy inhibition might represent a novel strategy to sensitize cancer cells’ response to salinomycin. 

## Results

### Salinomycin induces autophagy in osteoblastoma cells

**Figure 1:**
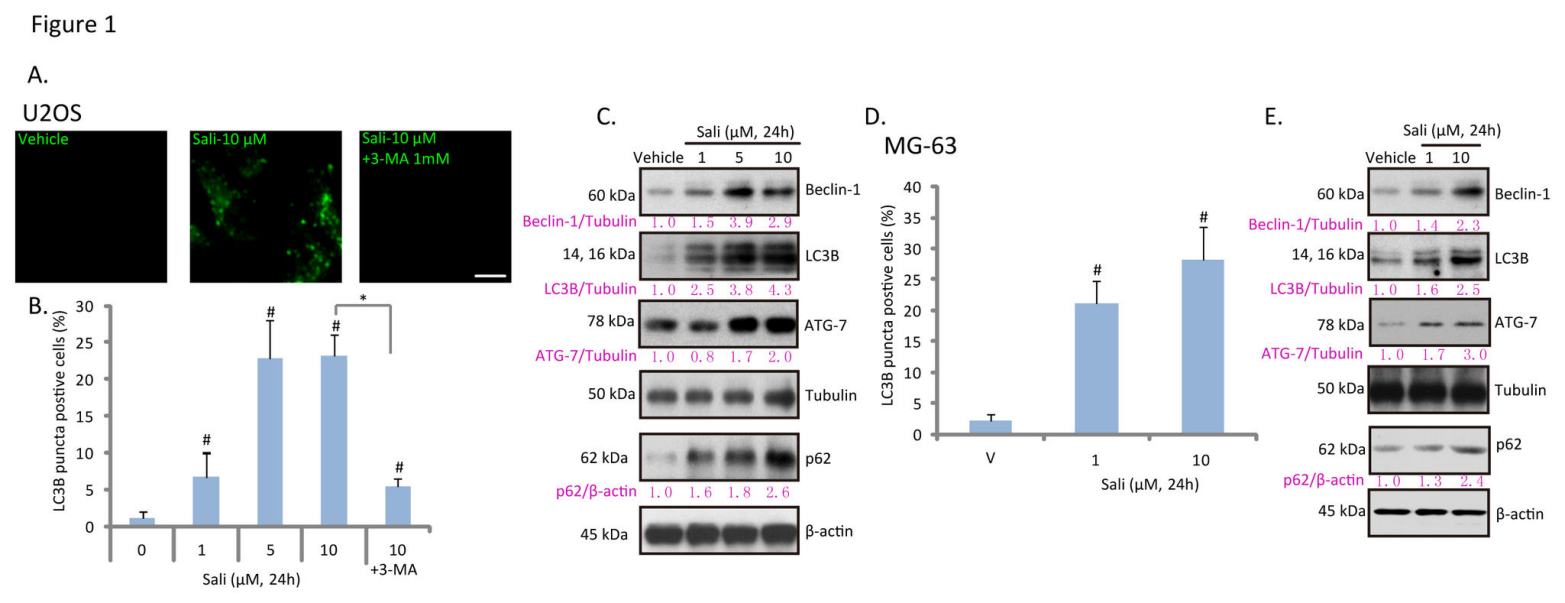
Salinomycin induces autophagy in osteoblastoma cells. Cultured U2OS osteoblastoma cells were treated with vehicle (0.1 % of DMSO), indicated concentration of salinomycin (Sali) or salinomycin (10 μM) plus 3-methyladenine (3-MA, 1 mM) for 24 hours, LC3B puncta fluorescence was detected by the confocal microscopy as described (A); the percentage of LC3B puncta positive cells was recorded (B), the expressions of LC3B, beclin-1, ATG-7, p62, β-actin and tubulin were detected by western blots (C). Cultured MG-63 cells were treated with vehicle or salinomycin (Sali, 1 and 10 μM) for 24 hours, LC3B puncta formation (D) and expressions of LC3B, beclin-1, ATG-7, p62, β-actin and tubulin were tested (E). Experiments in this figure were repeated three times. ^***#***^ *p*<0.05 vs. vehicle group. *p<0.05. Bar=15 μm (A).

The aim of this current study was to investigate the potential role of autophagy in salinomycin-induced cytotoxicity in cultured osteoblastoma cells, and to elaborate the underlying mechanisms. Autophagy starts with double membrane vesicles (autophagosomes) formation in the cytoplasm [16]. Autophagosomes degrade cytoplasmic material by acidic lysosomal hydrolases [22]. Microtubule-associated protein 1 light chain 3B (LC3B) is one of the key factors in autophagosome formation and autophagy initiation. LC3B is cleaved and conjugated to phosphatidylethanolamine to become LC3B-II, which forms pre-autophagosomal puncta structure [22]. As such, cleaved LC3B (LC3B-II) formation is detected as an key indicator of autophagy [22]. LC3B puncta immunofluorescence images in Figure 1A and quantified results in Figure 1B confirmed autophagy induction by salinomycin in U2OS cells, which was prevented by 3-MA, the autophagy inhibitor [23] (Figure 1A and B). The western-blot results in Figure 1C confirmed that LC3B-II (14 kDa), beclin-1 and ATG-7 were all upregulated by salinomycin in U2OS cells, further suggesting autophagy induction in these cells. The autophagy induction was also seen in salinomycin-treated MG-63 osteoblastoma cells, as the number of LC3B puncta positive cells and expressions of beclin-1/LC3B-II /ATG-7 were increased after salinomycin stimulation (Figure 1D and E). In the current study, an increase in p62 expression was seen in salinomycin-treated osteoblastoma cells (Figure 1C and E). p62 has emerged as a crucial molecule in autophagy, probably due to its ability to regulate several key steps of autophagy [24,25]. p62 shuttles the autophagic cargo to the autophagosome by directly binding with the autophagosomal membrane protein LC3 through the linear motif (LC3-interacting region) [26]. As such, p62 acts as an adaptor between ubiquitination of protein aggregates and the autophagy machinery degradation [26]. However, the increase of p62 by salinomycin could possibly result from its reduced degradation due to autophagy inhibition [27,28]. This is unlikely the case here. Since first, the mRNA expression of p62 was increased by salinomycin in U2OS cells (Figure S1A). More importantly, salinomycin-induced p62 expression was also seen in the presence of bafilomycin A1, the proteolysis and autophagy inhibitor that increased p62 by itself (Figure S1B). Further, our data generally supported autophagy activation, but not inhibition by salinomycin (Figure 1). 

### Autophagy inhibition enhances salinomycin-induced cytotoxicity in osteoblastoma cells

**Figure 2:**
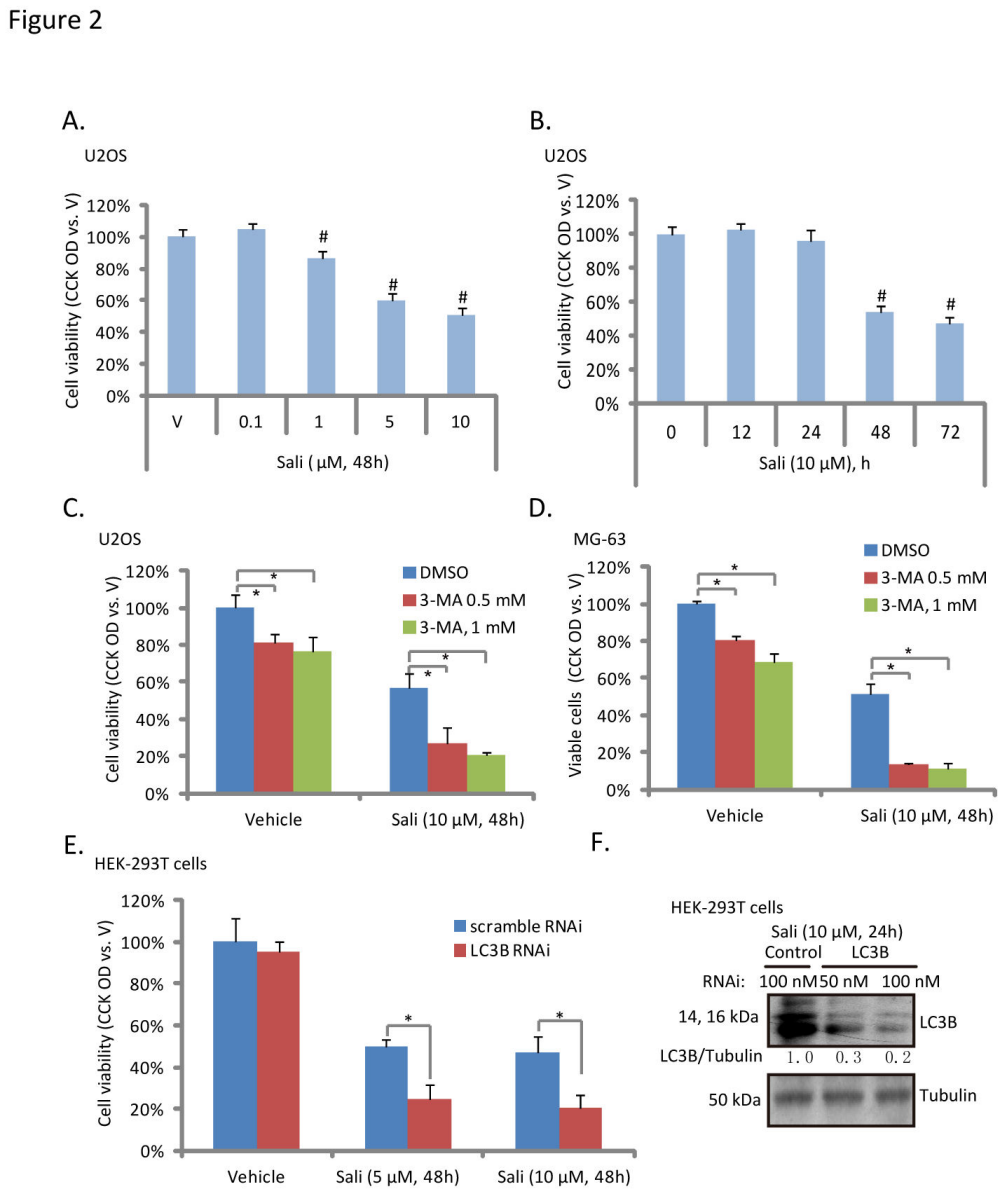
Autophagy inhibition enhances salinomycin-induced cytotoxicity in osteoblastoma cells. U2OS cells were treated with indicated concentration of salinomycin (Sali, 0.1, 1, 5 and 10 μM) for 48 hours (A), or treated with 10 μM of salinomycin for indicated time (B), cell viability was analyzed by CCK-8 cell viability assay. U2OS and MG-63 cells were treated with vehicle (0.1 % of DMSO) or salinomycin (Sali, 10 μM) in the presence or abscess of 3-MA (0.5 or 1 mM) for 48 hours, cell viability was analyzed by CCK-8 assay (C and D). Scramble RNAi- or LC3B siRNA-transfected HEK-293T cells were treated with salinomycin (Sali, 5 and 10 μM), cell viability was analyzed (E), and LC3B and tubulin expression was also examined to confirm the transfection efficiency (F). Experiments in this figure were repeated three times. ^***#***^ *p*<0.05 vs. vehicle (“V”) group. *p<0.05.

To test the potential role of autophagy in salinomycin-induced osteoblastoma cell cytotoxicity, we first examined salinomycin’s effect on U2OS cell viability. The CCK-8 assay results in Figure 2A and B showed that salinomycin inhibited U2OS cell viability in a dose- and time- dependent manner. Note that no significantly cell viability loss was observed with treatment of salinomycin at concentration lower than 1 μM (Figure 2A). Meanwhile, it took at least 48 hours for salinomycin (10 μM) to cause significant viability decrease or cell death (Figure 2B). Importantly, 3-MA, the autophagy inhibitor (Figure 1), enhanced salinomycin-induced U2OS and MG-63 cell viability loss (Figure 2C and D). Further, LC3B RNAi also enhanced salinomycin-induced cell viability loss in HEK-293T cells (Figure 2E and F). Notably, 3-MA as a single agent also induced moderate U2OS and MG-63 cell viability loss (Figure 2C and D). These results suggested that autophagy inhibition enhances salinomycin-induced osteoblastoma cell death.

### Inhibition of autophagy facilities salinomycin-induced apoptosis in osteoblastoma cells

**Figure 3:**
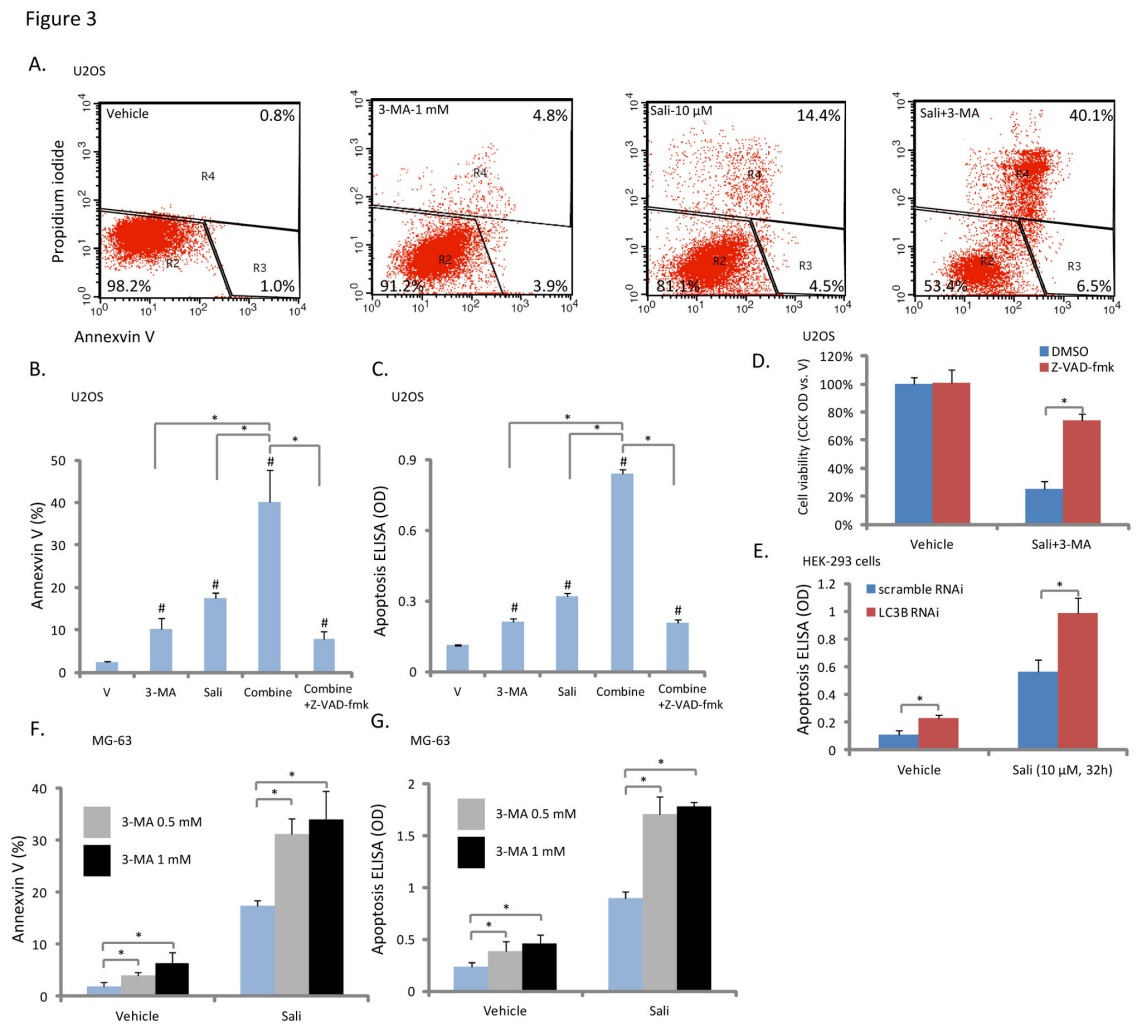
Autophagy inhibition facilities salinomycin-induced apoptosis in osteoblastoma cells. U2OS cells were treated with vehicle (0.1 % of DMSO), or 10 μM of salinomycin in the presence or abscess of 3-MA (1 mM) for 32 hours, Annexin V positive cells were sorted by FACS (A), quantified results were shown in (B). U2OS cell apoptosis was also detected by Histone-DNA ELISA assay as described (C), some of the cells were also pretreated z-VAD-fmk (50 μM) for 2 hours (B and C). U2OS cells were pre-treated with z-VAD-fmk (50 μM) for 2 hours, followed by salinomycin (10 μM) plus 3-MA (1 mM) co-administration, after 48 hours of culture, cell viability was analyzed (D). Scramble or LC3B siRNA (100 nM each) transfected HEK-293T cells were treated with salinomycin (Sali, 10 μM) for 32 hours, cell apoptosis was analyzed by histone-DNA ELISA assay (E). The effect of 3-MA (0.5 or 1 mM) on salinomycin (Sali, 10 μM, 32 hours)-induced MG-63 cell apoptosis was also shown (F and G). Experiments in this figure were repeated three times, similar results were obtained. *p<0.05.

While sustained and rigorous autophagy promotes cell apoptosis, mild or moderate autophagy is cell protective [29–31]. Above results have shown that autophagy inhibition by 3-MA or LC3B-RNAi enhanced salinomycin-induced cytotoxicity (Figure 2), we then tested if this was due to enhanced cell apoptosis. As shown in Figure 3A and B, the autophagy inhibitor 3-MA significantly enhanced salinomycin-induced apoptosis (more Annexin V positive cells) in U2OS cells. The histone-DNA ELISA assay results further confirmed apoptosis enhancement by 3-MA (Figure 3C). Note that 3-MA alone also induced minor cell apoptosis (Figure 3A-C). Z-VAD-fmk, the general caspase inhibitor, significantly suppressed 3-MA plus salinomycin-induced cell apoptosis (Figure 3B and C) and viability loss (Figure 3D), suggesting that autophagy inhibition enhanced salinomycin-induced cytotoxicity probably through facilitating cell apoptosis. Further, we found that LC3B RNAi knock-down also enhanced salinomycin-induced apoptosis in HEK-293 cells (Figure 3E). Meanwhile, in MG-63 cells, 3-MA also significantly enhanced salinomycin-induced cell apoptosis (Figure 3F and G). Thus, Inhibition of autophagy facilities salinomycin-induced apoptosis in osteoblastoma cells.

### Activation of AMPK mediates salinomycin-induced autophagy in osteoblastoma cells

**Figure 4:**
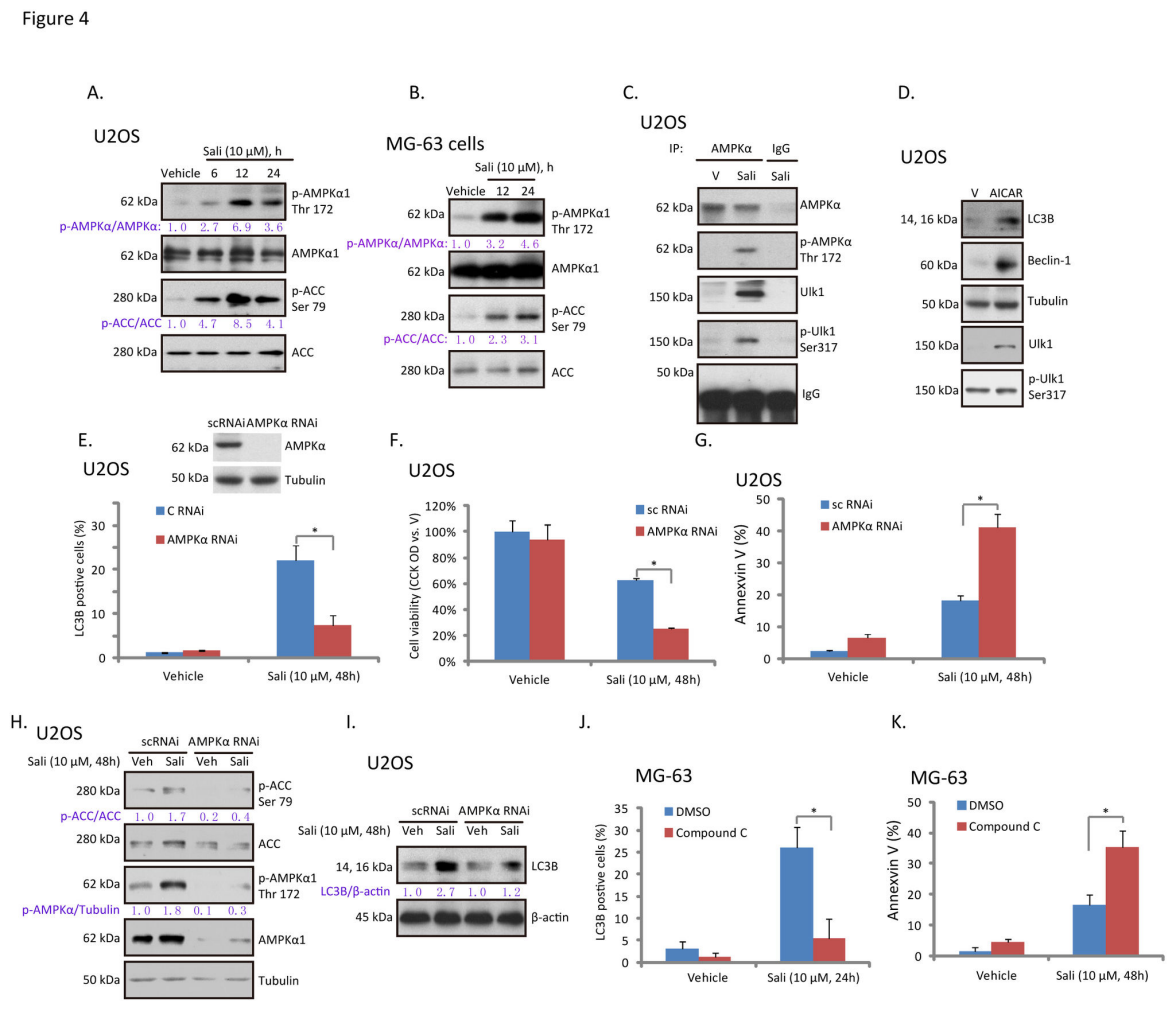
Activation of AMPK mediates salinomycin-induced autophagy in osteoblastoma cells. U2OS (A) and MG-63 (B) cells were treated with vehicle, or 10 μM of salinomycin (Sali), phospho (p)- and total AMPKα1 (Thr 172) and ACC (Ser 79) were examined by western blots. P- AMPKα and ACC were quantified. The association between AMPKα (total and p-) and Ulk1(total and p-) in vehicle-, or salinomycin (Sali, 10 μM, 12 hours)-treated U2OS cells were examined by co-IP (C). U2OS cells were treated with vehicle (“V”), or 1 mM of AICAR for 24 hours, p- and total Ulk1, LC3B-II, beclin-1 and tubulin were examined by western blots (D). Control or AMPKα stable knockdown U2OS cells were treated with 10 μM of salinomycin (Sali) for 48 hours, LC3B puncta (E), cell viability (F) and Annexin V percentage (G) were detected. Activation of AMPK (H) and the expressions of LC3B/β-actin (I) were also tested. The effect of compound C (25 μM, 1 hour pretreatment) on salinomycin (Sali, 10 μM)-induced autophagy and apoptosis were detected by LC3B puncta staining (J) and Annexin V sorting (K) in MG-63 cells respectively. Experiments in this figure were repeated three times. *p<0.05.

Next we focused on the underlying mechanism of autophagy induction by salinomycin. As discussed, activation of AMPK is important for autophagy induction [21,32]. We first examined AMPK activation in salinomycin-treated osteoblastoma cells. AMPK activation was reflected by AMPKα1 phosphorylation at Thr 172 and ACC phosphorylation at Ser 79. As demonstrated, a profound AMPK activation was observed in both U2OS and MG-63 cells after salinomycin treatment (Figure 4A and B), as AMPKα1 and ACC phosphorylation were both significantly increased (Figure 4A and B). Co-IP results in Figure 4C confirmed that salinomycin induced AMPK/Ulk1 association in U2OS cells, which appeared to cause Ulk1 phosphorylation. AICAR, the AMPK agonist [33], promoted Ulk1 phosphorylation and LC3B-II/beclin-1 expression in U2OS cells (Figure 4D). On the other hand, AMPKα1/2 RNAi stable-knockdown significantly inhibited salinomycin-induced autophagy in U2OS cells (Figure 4E), correspondingly, U2OS cell viability loss and apoptosis were increased (Figure 4F and G). Results in Figure 4H showed that, after 48 hours salinomycin stimulation, AMPK was still activated in U2OS cells, which were blocked by AMPKα RNAi. Further, salinomycin (10 μM, 48 hours)-induced LC3B expression was also suppressed by AMPKα stable knockdown in U2OS cells (Figure 4E). Similarly, compound C, the AMPK inhibitor, largely suppressed salinomycin-induced autophagy (Figure 4J), while enhancing apoptosis in MG-63 cells (Figure 4K). These results indicated that activation of AMPK by salinomycin mediates autophagy and apoptosis-resistance in osteoblastoma cells.

### Salinomycin-induced AMPK activation requires ROS production in osteoblastoma cells

**Figure 5:**
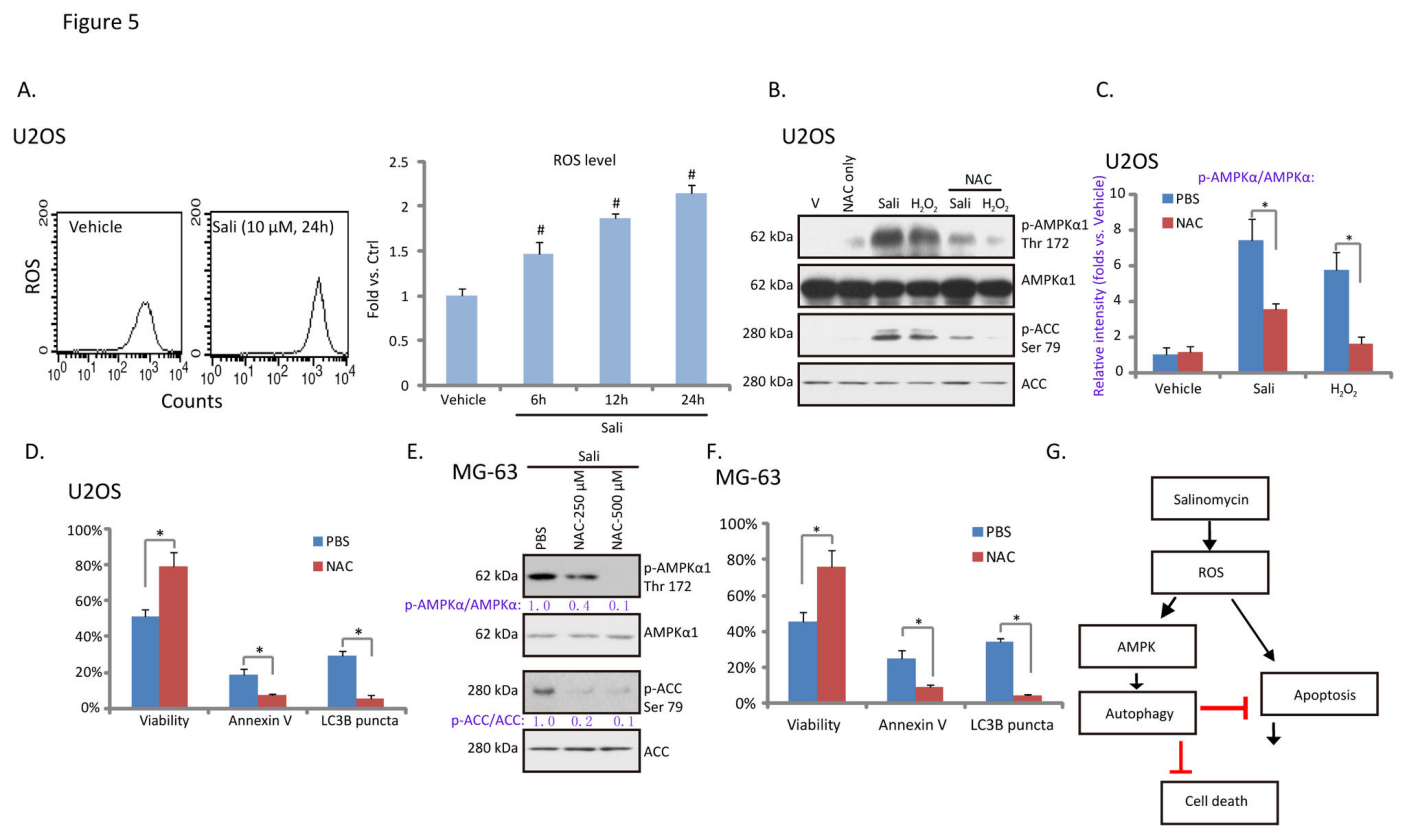
Salinomycin-induced AMPK activation requires ROS production in osteoblastoma cells. U2OS cells were treated with vehicle or 10 μM of salinomycin (Sali) for 6, 12 and 24 hours, cellular ROS level was analyzed, quantified results were shown (A). The effect of NAC (500 μM, 3 hours pretreatment) on salinomycin (Sali, 10 μM, 12 hours) or H_2_O_2_ (250 μM, 2 hours)-induced AMPK activation in U2OS cells was tested by western blots (B). AMPK phosphorylation was quantified (C). U2OS and MG-63 cells were treated with salinomycin (Sali, 10 μM) in the presence or absence of NAC (500 μM, 3 hours pretreatment), cell viability, Annexin V percentage, and LC3B puncta were also detected (D and F). The effect of NAC (250/500 μM, 3 hours pretreatment) on salinomycin (Sali, 10 μM, 12 hours)-induced AMPK activation in MG-63 cells was also tested (E). (G) The proposed signaling pathways of this study: in osteoblastoma cells, salinomycin induces ROS production, which is required for both AMPK activation and apoptosis. Activated AMPK promotes autophagy activation, which is anti-apoptosis and anti-cell death. Experiments in this figure were repeated three times. ^***#***^ *p*<0.05 vs. Vehicle group. *p<0.05.

Next we focused on the underlying mechanism of AMPK activation by salinomycin in osteoblastoma cells. Results in Figure 5A demonstrated the ROS production in salinomycin-treated U2OS cells, similar effect by salinomycin was also seen in other cell lines [8,34,35]. Significantly, the antioxidant NAC inhibited salinomycin-induced AMPK activation (Figure 5B and C). Meanwhile, hydrogen peroxide (H_2_O_2_) induced AMPK activation in U2OS cells (Figure 5B and C), which was also prevented by NAC pre-administration. Interestingly, NAC inhibited salinomycin-induced both autophagy and apoptosis (Figure 5D) in U2OS cells, while promoting cell survival (Figure 5D). These results indicate that ROS production by salinomycin might be required for both AMPK-autophagy activation and cell apoptosis (Figure 5G). We observed similar results in MG-63 cells, where NAC inhibited salinomycin-induced AMPK-autophagy activation, cell apoptosis and viability loss (Figure 5E and F). Together, these results suggested that salinomycin-induced AMPK activation requires ROS production.

## Discussion

Here we observed that salinomycin induced both apoptosis and autophagy in cultured osteoblastoma cells. Inhibition of autophagy by its inhibitor 3-MA, or by LC3B RNAi, enhanced salinomycin-induced cytotoxicity and apoptosis, indicating that autophagy induction by salinomycin was against cell death and apoptosis (Figure 5G). For the mechanism study, we proposed that AMPK activation by salinomycin mediated autophagy activation. AMPK inhibition by compound C or by stable RNAi suppressed salinomycin-induced autophagy in osteoblastoma cells, thus enhancing cell apoptosis and cytotoxicity (Figure 5G). We found that ROS production might be required for AMPK activation and cell autophagy by salinomycin. 

It has been shown by different groups that salinomycin activates autophagy in multiple cancer cell lines [34–36]. The underlying mechanisms, however, are not fully understood. Here, we discovered that salinomycin induced significant AMPK activation in cultured osteoblastoma cells, which promoted Ulk1 activation and autophagy initiation. Kim et al., has demonstrated a molecular mechanism for regulation of Ulk1, the autophagy trigger, by AMPK. AMPK activates autophagy by directly binding and activating Ulk1 through phosphorylation of Ser 317 and Ser 777. Following studies have identified other possible phosphorylation sites of Ulk1 by AMPK [10,37]. Reversely, autophagy is inhibited by the mammalian target of rapamycin (mTOR). mTOR phosphorylates Ulk1 at Ser 757 to lock Ulk1 into its complex, and stops Ulk1 from binding to AMPK [21,37]. In the current study, we found that salinomycin-activated AMPK directly associated and phosphorylated Ulk1 at Ser 317, which might be the key mechanism for autophagy induction. This proposal was further supported by the fact that compound C and AMPKα RNAi abolished salinomycin-induced autophagy, while AMPK agonist AICAR promoted autophagy in osteoblastoma cells. 

AMPK activation dictates energy metabolism, gene transcription, cell mitosis and autophagy through regulating its many downstream kinases [10,38]. However, the definitive role of AMPK in cell survival or apoptosis is still controversial. A number studies have found that sustained AMPK activation under severe stress conditions may inhibit cell growth and promote cancer cell apoptosis [39–41]. Others found that AMPK is pro-survival and anti-apoptotic [42,43]. One explanation is that, depends on intensity of the stress, AMPK might coordinate with other kinases to rescue cells when facing minor or moderate stresses, or to promote cell apoptosis when the rescue fails. In our system, we observed that AMPK activation by salinomycin was anti-cell apoptosis, while AMPK inhibition enhanced cytotoxicity and apoptosis by salinomycin in osteoblastoma cells. 

Here we provided evidence to support that ROS production is involved in AMPK activation by salinomycin. It is not surprising as first, salinomycin is known to induce ROS production in cancer cells [8], and second, ROS is well-known activator of AMPK [44–46]. The mechanism of salinomycin-mediated induction of oxidative stress is not clear. Recent studies described that salinomycin administration induces mitochondrial membrane potential decrease which might be responsible for ROS production and cancer cell apoptosis [8,47]. Others showed that production of H_2_O_2_ and O2^+^ after salinomycin treatment in cancer cells [34]. Nevertheless, the detailed mechanism responsible for ROS production by salinomycin needs further characterization. Interestingly, we found that ROS was not only required for AMPK-autophagy activation, it was also important for salinomycin-induced apoptosis and cell death .Thus we concluded that ROS production by salinomycin activates autophagy and apoptosis simultaneously, while apoptosis mediates cell death, autophagy serves as a negative feedback trying to rescue cells (Figure 5G). As a matter of fact, a recent study showed that AMPK is critical for NADPH (nicotinamide adenine dinucleotide phosphate) maintenance [43]. AMPK phosphorylates and inhibits ACC, thus decreasing NADPH consumption. Meanwhile, AMPK activation increases NADPH generation by means of fatty-acid oxidation [43]. Thus, AMPK activation by salinomycin could work as the oxidative stress scavenger to inhibit oxidative stress, and to rescue cells. 

In conclusion, we here found that salinomycin induces autophagy in osteoblastoma cells through AMPK activation, which serves as a negative regulator against cell apoptosis and death. AMPK-autophagy inhibition might represent a novel strategy for salinomycin chemo-sensitization in osteoblastoma and possible other cancer cells.

## Materials and Methods

### Chemicals and reagents

Salinomycin, 3-methyladenine (3-MA), hydrogen dioxide (H_2_O_2_), bafilomycin A1 and N-acetyl-cysteine (NAC) were purchased from Sigma (Sigma, St. Louis, MO). Lipofectamine^TM^ 2000 and Plus TM reagent were obtained from Invitrogen (Shanghai, China). The AMPK inhibitor compound C was purchased from Calbiochem (Shanghai, China). Antibodies of AMPKα1, acetyl-CoA carboxylase (ACC), p62, β-actin ,tubulin, beclin-1, rabbit/mouse horseradish peroxidase (HRP)-conjugated IgG were purchased from Santa Cruz biotechnology (Santa Cruz, CA). Anti-LC3B, autophagy-related genes 7 (ATG-7), β-actin, phospho-AMPKα (Thr 172) and phospho-ACC (Ser 79), p-Ulk1 (Ser 317) and Ulk1 antibodies were obtained from Cell Signaling Tech (Denver MA). The enhanced chemiluminescence (ECL) western blot reagent kit was purchased from Pierce (Rockford, IL). 

### Cell Culture

U2OS and MG-63 osteoblastoma cells as well as HEK-293T were gifts from Dr. Zhang-Ping Gu at Nanjing Medical University [48,49], cells were maintained in DMEM (Sigma, St. Louis, MO), supplemented with a 10% FBS (Sigma) plus penicillin/streptomycin (1:100; Sigma), in a CO_2_ incubator at 37 °C. 

### Western blot and data quantification

After indicated treatments, the cells were washed with ice-cold phosphate buffered saline (PBS) and then lysed using lysis buffer (pH, 7.4) containing 50 mM Tris [pH 8.0], 250 mM NaCl, 1% NP-40, 0.1% sodium dodecyl sulfate, 5 mM EDTA, 2 mM Na_3_VO_4_, 10 mM Na_2_P_2_O_7_, 10 mM NaF and 1 mM phenylmethylsulfonyl fluoride. The lysates were collected and centrifuged. The concentration of the extracted protein samples was measured by protein concentration assay kit (Sigma-Aldrich). The extracted protein sample was boiled for 5 min in 5-times loading buffer. Samples were separated by 10% SDS-polyacrylamide gel, and electro-transferred onto a polyvinylidene fluoride (PVDF) membrane (Millipore, USA). Afterwards, the membrane was blocked with blocking buffer (10% (w/v) milk in PBS Tween-20 (PBST), incubated overnight at 4 °C with the indicated primary antibody, and then incubated with HRP-conjugated second antibody at room temperature for 1-2 hours. The detection was performed by ECL Supersingnal West Pico Chemiluminescent Substrate according to the manufacturer’s instruction. The intensity of indicated band was quantified by densitometry using ImageJ software, and was normalized to non-phosphorylated kinase or the loading control. Quantification value was expressed as the fold change vs. the band labeled with “1.0”. ImageJ was downloaded from NIH website (http://rsbweb.nih.gov/ij/download.html).

### CCK-8 cell viability assay

After treatment, the cell viability was measured by Cell Counting Kit-8 (CCK-8) (Dojindo, Japan) assay according to manufacturer’s protocol [50]. The OD value of the group received the indicated treatment was normalized to OD value of vehicle-treated control group. Loss of cell viability was used as the indicator of cell death in this study.

### Analysis cell apoptosis by flow cytometry detecting FITC-Annexin V positive cells

Cell apoptosis was detected by the Annexin V Apoptosis Detection Kit (Biyuntian, Shanghai, China) according to the manufacturer’s protocol. Briefly, cells with indicated treatment were stained with FITC-Annexin V and propidium iodide (PI) (Biyuntian, Shanghai, China). Both early (Annexin V^+^/PI^−^) and late (Annexin V/PI^+^) apoptotic cells were sorted by fluorescence-activated cell sorting (FACS) (Beckman Coulter, Inc., Brea, CA). Cell apoptosis was reflected Annexin V percentage.

### Quantification of apoptosis by enzyme-linked immunosorbent assay (ELISA)

The Cell Apoptosis Histone-DNA ELISA Detection Kit (Roche, Palo Alto, CA) was utilized to further test cell apoptosis, according to the manufacturer's protocol. Briefly, the cytoplasmic histone/DNA fragments from cells were extracted and bound to the immobilized anti-histone antibody (attached in the kit). Subsequently, the peroxidase-conjugated anti-DNA antibody was added for the detection of immobilized histone/DNA fragments. After addition of substrate for peroxidase, the spectrophotometric absorbance of the samples was determined using a plate reader at a test wavelength of 405 nm. OD value was utilized as indicator of the extent of apoptosis induction [51].

### LC3B RNA Interference (RNAi) in HEK-293T cells

SiRNA for LC3B was purchased from Cellular Signaling Tech (*SignalSilence® LC3B siRNA I #6212*). HEK-293T cells were cultured in regular growth medium containing no antibiotic, and were seeded in a six-well plate with 60% of confluence. For RNAi experiments, 2.0 μl PLUS™ reagent (Invitrogen, Carlsbad, CA) was diluted into 90 μl of RNA dilution water (Santa Cruz) for 5 min at room temperature. Then, 10 μl of siRNA (scramble or LC3B) was added to PLUS™ reagent for 5 min at room temperature. Lipofectamine^TM^ (2.0 μl) (Invitrogen) was then added to the complex. After 30-min incubation at room temperature, the transfection complex was formed and added to each well containing 1 ml of transfection medium (no antibiotic, no FBS) with the final LC3B/scramble siRNA concentration of 50-100 nM. Growth medium (10% FBS, no antibiotic) were switched back to HEK-293T cells 8 hours after transfection, and cells were further cultured for additional 24 hours. LC3B expression in transfected cells was determined by western blot to insure RNAi efficiency, only LC3B knockdown cells were used for further experiments.

### LC3B immunochemistry

U2OS and MG-63 cells were grown as monolayer on cover-slips, cells were fixed in cold formalin for 15 min at -4 °C, washed three times with PBS, and blocked with 5% BSA in PBS (pH 7.5) for 30 min, followed by overnight incubation with the primary antibody (anti-LC3B, Cell Signaling Tech, 1:25) at 4 °C. The next day, the secondary fluorescent FITC (Biyuntian, Shanghai, China) at dilution of 1:100 was added. Cells were visualized by using a Leica microscope fitted with the appropriate filters. The percentage LC3B puncta positive cells (green fluorescence) was recorded. Experiments were repeated four times, each with five wells. For each count, a total of at least 200 cells in a view from independent treatment were counted.

### Co-Immunoprecipitation (Co-IP)

Cell lysates (1000 μg) in 1 mL lysis buffer containing 1% Triton and 0.3% CHAPS were pre-cleared with 30 μl of protein IgA/G-beads (Sigma) for 30 min at 4 °C. After centrifugation for 10 min at 4 °C in a micro-centrifuge, the supernatant was rotated overnight with 2 μg of indicated primary antibody (anti-AMPKα1/2, Santa Cruz). Protein IgA/G-beads (35 μl) were added to the supernatants for 2 h at 4 °C. Then the pellets were washed six times with lysis buffer buffer, resuspended in lysis buffer, and then assayed in western-blots to detect phospho- and total-Ulk1 and AMPKα. 

### Generation of stable AMPKα knockdown U2OS cells by lentiviral transfection

U2OS cells were seeded in a six-well plate with 60% of confluence in growth medium without antibiotic. 10 μl/milliliter of lentiviral particles containing AMPKα shRNA (a gift from Dr. Qing-you Zheng [41]) were added to the cells for 24 hours, afterwards, cell medium was replaced by fresh growth medium for another 24 hours. Cells were further cultured in puromycin (1 μg/ml)-containing growth medium, until resistant colonies can be identified. The expression of AMPKα1 in stable cells was always checked by western blots in the resistant colonies. Same amount of scramble shRNA lentiviral particles (Santa Cruz) was added into control cells.

### Measurement of intracellular reactive oxygen species (ROS)

Intracellular ROS production was measured using a DCFH-DA fluorescent dye (Molecular Probes/Invitrogen). U2OS cells were cultured in six-well plates at a density of 1*10^5^/well. After treatment, cells were incubated with 10 μM of DCFH-DA at 37 °C for 20 min and then washed twice with PBS. Cells were then analyzed for fluorescence using the flow cytometer mentioned above. The ROS level in the treatment group was normalized to that of control group. Experiment were repeated four times to calculate mean. 

### RT-PCR

Total RNA was extracted from U2OS cells with SV total RNA purification system (Promega, Shanghai, China) according to manufacturer’s protocol. A 2-µg of total RNA was reverse-transcribed using the reverse transcriptase (Promega, Madison, WI, USA). cDNA derived from 0.5 µg of total RNA was amplified by real-time polymerase chain reaction (PCR). SYBR Green PCR kit (Applied Biosystems, Foster City, CA) was used to detect p62 and glyceraldehyde phosphate dehydrogenase (GAPDH) expression. Primer sequences were as follows. For GAPDH; F: 5’-GAAGGTGAAGGTCGGAGTC-3’, R: 5’-GAAGATGGTGATGGGATTTC-3’. For p62; F: 5’-CTGCCCAGACTACGACTTGTGT-3’ and R: 5’-TCAACTTCAATGCCCAGAGG-3’ [52]. PCR was performed in triplicate and was conducted using a Real-Time PCR Detection System (7500; ABI, Carlsbad, CA, USA). The PCR data were analyzed. MRNA levels were normalized relative to GAPDH value. Fold expression changes and standard deviations (SD) were calculated. Three replicate reactions per sample and endogenous control were used to ensure statistical significance.

### Statistical analysis

The data presented were mean ±standard deviation (SD). Statistical differences were analyzed by one-way ANOVA followed by multiple comparisons performed with post hoc Bonferroni test (SPSS version 16). Values of *p*<0.05 were considered statistically different. 

## Supporting Information

Figure S1 **(A) RT-PCR analysis of p62 and GAPDH mRNA in U2OS cells after indicated salinomycin stimulation.** (B) Western blot analysis of p62 and β-actin in U2OS cells after indicated treatment.
Experiments in this figure were repeated three times and similar results were obtained.
(EPS)
